# Effects of a family collaboration care model on disease perception, self-management, and quality of life in elderly patients with multidrug-resistant pulmonary tuberculosis: a retrospective study

**DOI:** 10.3389/fmed.2026.1781223

**Published:** 2026-06-03

**Authors:** Zhaojie Wang, Jing Pang

**Affiliations:** Henan Provincial Chest Hospital, Zhengzhou University, Zhengzhou, China

**Keywords:** disease perception, family collaboration care model, multidrug-resistant pulmonary tuberculosis, quality of life, self-management ability

## Abstract

**Objective:**

This retrospective study evaluated the impact of a family collaboration care model on disease perception, self-management, and quality of life among elderly patients with multidrug-resistant pulmonary tuberculosis (MDR-TB).

**Methods:**

A retrospective study was conducted on elderly patients diagnosed with MDR-TB at Henan Provincial Chest Hospital between January 2022 and December 2024. Based on nursing records, patients were classified into a standard care group and a family collaboration care group. Disease perception, self-management ability, and quality of life were assessed using the BIPQ, AHSMSRS, and WHOQOL-BREF, respectively. The partial eta-squared, Cohen’s d, and Hedges’ g were used to evaluate effect size.

**Results:**

A total of 57 elderly patients (45 males, 12 females) were included, with 30 in the standard care group and 27 in the family collaboration care group. Compared with the standard care group, the family collaboration care group had significantly lower total disease perception scores (33.44 ± 4.82 vs. 40.97 ± 7.10, *p* < 0.001), higher self-management scores (131.85 ± 13.97 vs. 108.13 ± 11.29, *p* < 0.001), and higher quality-of-life scores (*p* < 0.001 for all domains). Effect sizes were large for disease perception (Hedges’ g = −1.211), self-management ability (Hedges’ g = 1.856), and quality of life (Hedges’ g = 2.496).

**Conclusion:**

Family collaboration care may be associated with better disease perception, improved self-management ability, and enhanced quality of life among elderly patients with MDR-TB. This model may serve as a practical approach for optimizing nursing management in MDR-TB care, although its effectiveness warrants further validation in future studies.

## Introduction

Multidrug-resistant pulmonary tuberculosis (MDR-TB) is a severe form of tuberculosis caused by *Mycobacterium tuberculosis* strains resistant to at least isoniazid and rifampicin, the two most potent anti-tuberculosis drugs. MDR-TB poses a major public health challenge due to its complex treatment, prolonged therapy, high recurrence rate, and substantial mortality (1, 2). Studies have reported that the drug resistance rate among elderly tuberculosis patients can be high in certain populations or regions, with a multidrug resistance rate that is often lower than in younger or previously treated patients in many global contexts (with global averages for new cases around 3–4%) (3, 4). However, the highest mortality is consistently observed among older individuals, particularly those aged 60 to 80, due to factors like existing health conditions and treatment challenges (5). MDR-TB poses a major public health challenge due to its complex treatment, prolonged therapy, high recurrence rate, and substantial mortality. The elderly population is particularly vulnerable, as they often present with multiple comorbidities, diminished immune function, and poor treatment tolerance (3, 6). Therefore, providing guidance on how elderly patients should respond to their illness is critically important for managing the health of MDR-TB patients, underscoring the urgent need for effective, patient-centered management strategies to improve outcomes in this high-risk group.

Disease perception refers to an individual’s cognitive and emotional understanding of their illness, influencing how they interpret symptoms, cope with treatment, and engage in self-care behaviors (7). A positive and accurate perception can promote treatment adherence, emotional stability, and recovery, whereas a negative disease perception often leads to fear, avoidance behaviors, and reduced quality of life. In elderly patients with MDR-TB, inaccurate or pessimistic illness perceptions have been associated with poor medication adherence, decreased self-management ability, and worse health outcomes (8). Therefore, helping patients to correctly understand and manage their disease perception is an important aspect of improving both psychological well-being and clinical prognosis.

Family support plays a crucial role in influencing patients’ emotional resilience, treatment confidence, and self-management behaviors (9). The family collaboration care model integrates patients and their family members into the nursing process, emphasizing shared responsibility, emotional support, and joint participation in care activities (10). This model encourages family members to assist in monitoring and guiding patients while working in partnership with professional healthcare staff to provide holistic care. Evidence has shown that such collaborative approaches can enhance patients’ satisfaction, adherence, and overall recovery experience (11). In China, existing research on disease perception in MDR-TB patients has mainly focused on identifying influencing factors, with limited exploration of structured, family-based nursing strategies to improve perception and self-management (12, 13). However, there is limited evidence on structured family-based interventions in elderly MDR-TB patients. Therefore, this study applied the family collaboration care model to evaluate its impact on disease perception, enhancing self-management abilities, and improving quality of life among elderly patients with MDR-TB.

## Methods

### Study design and patients

This retrospective study included elderly patients with pulmonary tuberculosis who were admitted to Henan Provincial Chest Hospital between January 2022 and December 2024. The study was reviewed and approved by the hospital’s Medical Ethics Committee (approved No.: (2024) Research Review (09–05)), and informed consent was waived due to the retrospective design.

Patients were eligible if they met the following inclusion criteria: (1) Diagnosis of pulmonary tuberculosis according to the “*Diagnosis and Treatment Guidelines for Pulmonary Tuberculosis*” (14), confirmed by clinical manifestations, radiographic findings, and bacteriological tests; (2) presence of multidrug-resistant tuberculosis, defined as resistance to at least isoniazid and rifampicin; (3) age ≥ 60 years, with family members aged > 18 years who participated in care. Exclusion Criteria were as follows: (1) comorbid malignant tumors, major organ failure, or other severe diseases. (2) acute or critical illness. (3) incomplete clinical or nursing data.

Due to the rarity of elderly MDR-TB cases and the retrospective design, this study included all eligible patients during the study period without a pre-specified sample size calculation. The final sample represents the maximum available cohort with complete data. Finally, although all participants met uniform inclusion and exclusion criteria, unmeasured confounding may still bias the observed associations.

### Grouping and care measures

**Figure 1:**
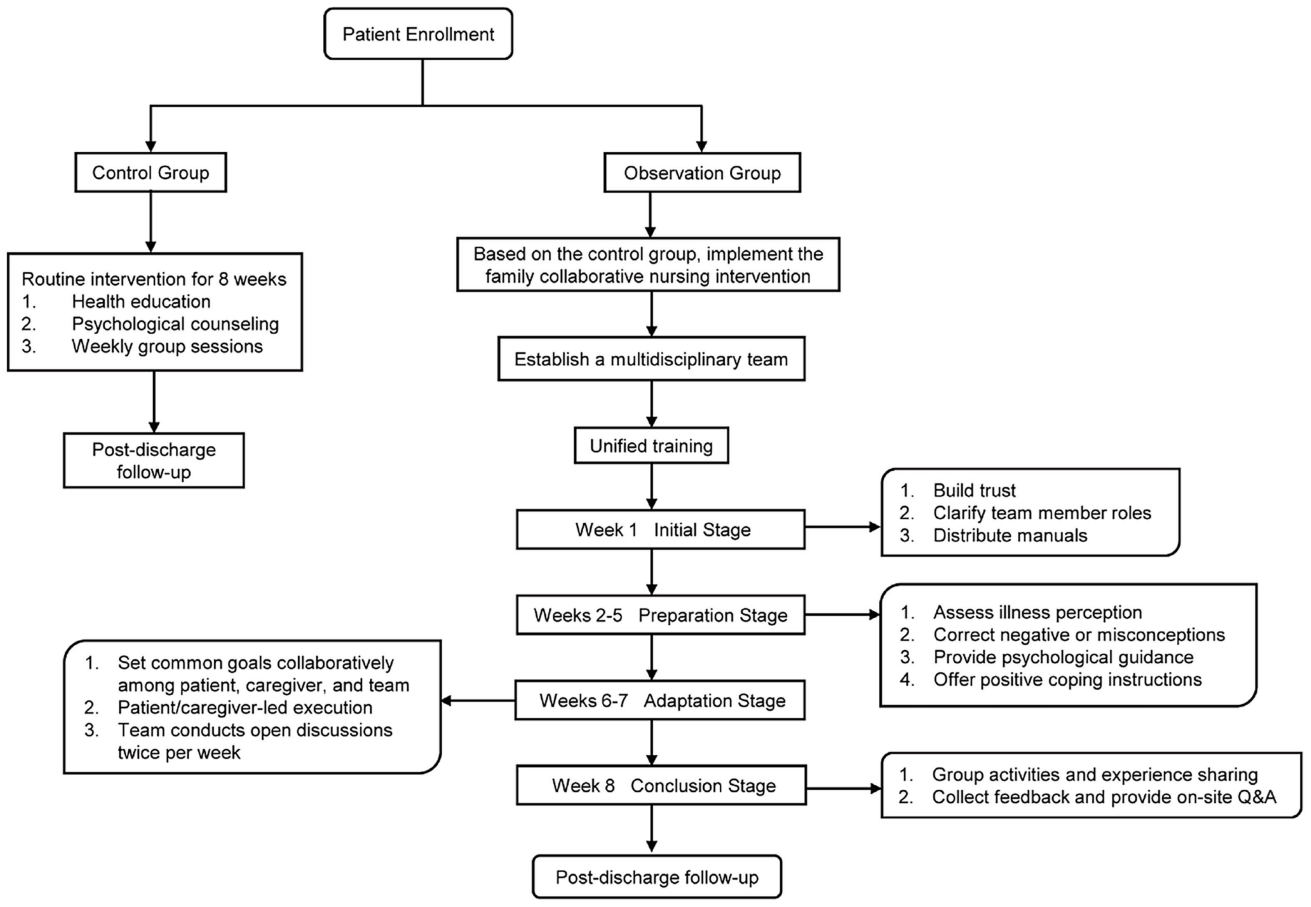
Flowchart of patient grouping and nursing interventions.

Based on the nursing records, patients were classified into a standard care group and a family collaboration care group according to the type of nursing care they received during hospitalization (Figure 1).

### Standard care group

Patients in the standard care group received routine nursing management provided by the healthcare team. Care measures included: (1) Education on disease-related knowledge, such as etiology, transmission, and medication guidelines. (2) Attention to psychological health, including counseling on simple methods to alleviate negative emotions, such as music therapy or communication with peers. (3) Weekly group health education sessions (30–40 min) conducted during hospitalization to address patient concerns. After discharge, follow-up was provided via telephone to remind patients of clinic visits and reinforce medication adherence.

### Family collaboration care group

Patients in the family collaboration care group received comprehensive nursing management based on the family collaboration care model in addition to standard care. This model emphasized active involvement of both patients and family caregivers in care delivery. A multidisciplinary team, comprising the head nurse (team leader), attending physician, primary nurses, pharmacist, and psychologist, was responsible for coordinating patient care. All team members underwent standardized training before implementation to ensure consistency of nursing practices.

The family collaboration care process was structured into four stages, each lasting approximately 2 weeks, for a total duration of 8 weeks:

*Initial Phase (Week 1)*: A trust relationship was established with patients and their primary caregivers. The objectives and procedures of the family collaboration care model were explained, emphasizing the caregivers’ role in recovery. Educational materials on MDR-TB and disease perception were provided to enhance health literacy.
*Preparation Phase (Weeks 2–5)*: The care team conducted open discussions with patients and caregivers to assess disease perceptions and identify inaccurate or negative beliefs. Targeted education and psychological guidance were provided to foster positive cognitive and emotional responses toward the disease. Caregivers were instructed to assist patients in medication management, diet regulation, and emotional support.
*Adaptation Phase (Weeks 6–7)*: As patients and caregivers developed a basic understanding of the disease, the care process transitioned to a more self-directed model. Together with the healthcare team, individualized goals were established to promote self-management and positive coping behaviors. The team maintained contact twice weekly through face-to-face or telephone consultations to provide feedback and address difficulties encountered in daily management.
*Consolidation Phase (Week 8)*: Group meetings were organized to allow patients and caregivers to share experiences, strengthen mutual support, and consolidate learned skills. The care team collected feedback and provided additional guidance for unresolved challenges to ensure the continuity of family-based collaborative care after discharge.

### Outcome

Disease perception was evaluated using the Brief Illness Perception Questionnaire (BIPQ) (14, 15), a validated tool assessing patients’ cognitive and emotional of illness. The BIPQ includes 9 items across three dimensions—cognitive, emotional, and understanding. Items 1–8 are scored on a scale of 0–10, while item 9 is an open-ended qualitative question and excluded from scoring. Items 3, 4, and 7 are reverse-scored. Higher total scores indicate more negative illness perceptions, greater severity of symptoms, and a more pessimistic understanding of the disease.

Self-management ability was assessed using the Adult Health Self-Management Scale (AHSMSRS) (16, 17), which comprises three dimensions: self-management environment, self-management cognition, and self-management behavior. Each item is rated on a 5-point Likert scale (1–5 points), with higher scores reflecting stronger self-management capability.

Quality of life was measured using the World Health Organization Quality of Life Brief Scale (WHOQOL-BREF) (18, 19). This scale includes 26 items, of which the first two questions assess overall quality of life and health satisfaction, and the remaining 24 items are divided into four domains: physical, psychological, social, and environmental. Each item is scored using a 5-point Likert scale (1–5 points), with domain scores converted to 4–20 points, and higher scores denote better perceived quality of life.

In the present cohort, the Cronbach’s alpha coefficients were 0.79 for the BIPQ, 0.80 for the AHSMSRS, and 0.809 for the WHOQOL-BREF. All three instruments were validated Chinese versions; therefore, no additional translation or cultural adaptation was performed in this study.

Also, baseline demographic and clinical data were extracted from electronic medical records. Variables included age, sex, body mass index (BMI), income, duration of disease, family support level, and education level. Family support scores were derived from nursing documentation assessing the degree of family involvement in care (scored 1–5).

### Statistical analysis

All statistical analyses were performed using SPSS version 25.0 (IBM Corp., Armonk, NY, United States). Continuous variables were tested for normality using the Kolmogorov–Smirnov test. Data conforming to a normal distribution were expressed as mean ± standard deviation (SD) and compared between groups using the independent-samples t-test. Non-normally distributed continuous variables were presented as median (interquartile range, P25-P75) and analyzed using the Mann–Whitney U test. Categorical variables were expressed as frequency and percentage [n (%)] and compared between groups using the chi-square test or Fisher’s exact test when appropriate.

To examine the effects of the intervention over time, a mixed-design ANOVA was conducted for each primary outcome. The interaction effect was evaluated using partial eta-squared (partial *η*^2^), with values of 0.01, 0.06, and 0.14 conventionally interpreted as small, medium, and large effects, respectively. Additionally, between-group effect sizes were estimated using both Cohen’s d and Hedges’ g. Positive values indicate higher scores in the intervention group, while negative values indicate lower scores relative to the control group. Effect magnitudes were interpreted using Cohen’s conventional thresholds (|d| ≥ 0.8 = large effect). All tests were two-tailed, and a *p* value < 0.05 was considered statistically significant.

## Results

### Baseline characteristics

A total of 57 elderly patients with MDR-TB were included in this study, comprising 30 patients in the standard care group and 27 patients in the family collaboration care group. Results showed that the general demographic and clinical characteristics are comparable between patients of the two groups (all *p* > 0.05; Table 1). There were no statistically significant differences between groups in age (69.20 ± 7.98 vs. 68.78 ± 8.36 years), gender distribution (male: 80.0% vs. 77.8%), BMI (21.94 ± 1.78 vs. 22.84 ± 2.16 kg/m^2^), monthly income (1835.00 ± 730.75 vs. 1885.19 ± 666.05 CNY), duration of disease (5.27 ± 1.78 vs. 5.59 ± 1.95 years), family support score (2.53 ± 1.46 vs. 2.41 ± 1.34), or education level (all *p* > 0.05).

**Table 1:** General characteristics of patients in the two groups.

Characteristics	Standard care group (*n* = 30)	Family collaboration care group (*n* = 27)	Statistic	*p*
Age, years	69.20 ± 7.98	68.78 ± 8.36	*t* = −0.195	0.846
Gender			*χ*^2^ = 0.042	0.837
Male	24	21		
Female	6	6		
BMI	21.94 ± 1.78	22.84 ± 2.16	*t* = 1.728	0.090
Income, CNY	1835 ± 730.75	1885.19 ± 666.05	*t* = 0.270	0.788
Duration of disease	5.27 ± 1.78	5.59 ± 1.95	*t* = 1.728	0.512
Family support	2.53 ± 1.46	2.41 ± 1.34	*χ*^2^ = 0.550	0.736
Education level			*χ*^2^ = 2.062	0.344
Primary school or below	7	3		
Middle school	8	9		
High school or technical college	10	8		
College or above	5	7		
Number of comorbidities	1.80 ± 0.925	1.67 ± 1.177	−0.478	0.635

### Disease perception

As shown in Table 2, there were no significant differences in disease perception scores between the two groups before standard or family collaboration care (all p > 0.05). After care, patients in the family collaboration care group demonstrated significantly lower total disease perception scores (33.44 ± 4.82 vs. 40.97 ± 7.10, *p* < 0.001). The interaction effects also showed a large effect (partial *η*^2^ = 0.510, *p* < 0.001), while the Cohen’s d and Hedges’g were −1.227 and −1.211, respectively. Across cognitive, emotional, and understanding dimensions, the family collaboration care group consistently outperformed the standard care group (all *p* < 0.01).

**Table 2:** Comparison of disease perception scores before and after intervention.

Group		Family collaboration care group (*n* = 27)	Standard care group (*n* = 30)	*p*
Cognitive	Pre-care	30.52 ± 5.90	30.83 ± 5.44	0.835
	Post-care	21.70 ± 3.82	26.07 ± 5.38	0.001
Emotional	Pre-care	12.52 ± 2.31	12.53 ± 1.99	0.979
	Post-care	7.85 ± 1.70	10.23 ± 1.78	<0.001
Understanding	Pre-care	5.93 ± 1.44	5.47 ± 1.50	0.245
	Post-care	3.89 ± 1.01	4.67 ± 1.16	0.009
Total score	Pre-care	48.96 ± 6.62	48.83 ± 5.26	0.935
	Post-care	33.44 ± 4.82	40.97 ± 7.10	<0.001

### Self-management ability

According to Table 3, no significant differences in self-management ability were observed between groups before standard or family collaboration care (all *p* > 0.05). After care, the family collaboration care group exhibited significantly higher total self-management scores (131.85 ± 13.97 vs. 108.13 ± 11.29, *p* < 0.001). The interaction effects also showed a large effect (partial *η^2^* = 0.498, *p* < 0.001), while the Cohen’s d and Hedges’g were 1.882 and 1.856, respectively. Improvements were also evident in all three dimensions (all *p* < 0.001).

**Table 3:** Comparison of self-management ability scores before and after intervention.

Group		Family collaboration care group (*n* = 27)	Standard care group (*n* = 30)	*p*
Self-management environment	Pre-care	26.19 ± 5.17	26.13 ± 5.18	0.97
	Post-care	33.78 ± 4.68	29.30 ± 4.19	<0.001
Self-management cognition	Pre-care	33.67 ± 4.57	33.63 ± 4.02	0.977
	Post-care	49.63 ± 5.39	40.40 ± 4.48	<0.001
Self-management behavior	Pre-care	30.37 ± 5.26	29.33 ± 3.60	0.395
	Post-care	48.44 ± 5.19	38.43 ± 4.31	<0.001
Total score	Pre-care	90.22 ± 10.38	89.10 ± 9.46	0.671
	Post-care	131.85 ± 13.97	108.13 ± 11.29	<0.001

### Quality of life

As shown in Table 4, quality-of-life scores did not differ significantly between groups before standard or family collaboration care (all *p* > 0.05). After care, the family collaboration care group demonstrated significantly higher quality-of-life scores across all four domains of the WHOQOL-BREF compared with the standard care group. Specifically, the physical (15.11 **±** 1.93 vs. 12.20 **±** 1.61, *p* < 0.001), psychological (16.07 **±** 1.66 vs. 13.13 ± 2.03, *p* < 0.001), social (16.26 **±** 1.35 vs. 13.67 **±** 1.47, *p* < 0.001), and environmental (15.85 **±** 1.70 vs. 12.73 **±** 1.68, *p* < 0.001) domain scores were all markedly improved in the family collaboration care group. For the total score, the interaction effect exhibited a large effect (partial *η*^2^ = 0.597, *p* < 0.001), while the Cohen’s d and Hedges’g were 2.531 and 2.496, respectively.

**Table 4:** Comparison of quality of life scores before and after intervention.

Group		Family collaboration care group (*n* = 27)	Standard care group (*n* = 30)	*p*
Physical	Pre-care	9.44 ± 2.06	9.13 ± 2.15	0.58
	Post-care	15.11 ± 1.93	12.20 ± 1.61	<0.001
Psychological	Pre-care	8.63 ± 1.50	8.40 ± 1.96	0.624
	Post-care	16.07 ± 1.66	13.13 ± 2.03	<0.001
Social	Pre-care	10.56 ± 1.91	10.07 ± 1.80	0.324
	Post-care	16.26 ± 1.35	13.67 ± 1.47	<0.001
Environmental	Pre-care	9.22 ± 1.91	9.30 ± 1.37	0.859
	Post-care	15.85 ± 1.70	12.73 ± 1.68	<0.001

## Discussion

This study demonstrated that the family collaboration care model significantly improved disease perception, self-management ability, and quality of life among elderly patients with MDR-TB. These findings suggest that involving family members in the care process, through the provision of personalized and targeted support, can enhance the psychological and physical well-being of MDR-TB patients, ultimately improving treatment adherence and clinical outcomes.

The results of this study are consistent with a growing body of research that underscores the positive impact of family-centered care models in managing chronic diseases. A substantial body of evidence supports the role of family involvement in improving disease perception, psychological well-being, self-management abilities, and overall health outcomes. For instance, studies in post-hepatectomy patients have shown that family support reduces anxiety and depression while enhancing the patient’s ability to manage their health effectively (20, 21). Similarly, research focusing on first-time mothers has highlighted the positive effects of family collaboration in improving mental health outcomes and self-management during the post-partum period (22).

In the context of chronic diseases, including TB, family-based care has proven beneficial in enhancing treatment adherence and quality of life. Research supports these findings, noting that family involvement can significantly improve psychological outcomes and clinical measures in chronic disease patients (23–25). Yet these studies rarely examined elderly patients with MDR-TB, a group for whom treatment is particularly burdensome because of lengthy and complex regimens, comorbidities, and high social stigma (26, 27).

The relevance of family collaboration in improving psychological and clinical outcomes is grounded in the biopsychosocial model of health, which emphasizes the interconnectedness of biological, psychological, and social factors in the management of health (28, 29). The family collaboration care model aligns with this framework by addressing not only the physical care needs of MDR-TB patients but also providing critical emotional and social support. An Indian study also reported that compared to hospitalization, decentralized treatment could significantly reduce costs but achieve similar outcomes (30). This approach fosters a sense of empowerment and active involvement in the treatment process, which is crucial for improving both adherence to treatment and overall quality of life (31). Our findings support the notion that family dynamics play a pivotal role in influencing health outcomes, particularly in elderly patients who may feel isolated or overwhelmed by their illness.

Despite the promising results, there are discrepancies between our findings and some previous studies that reported only modest improvements in the quality of life following family-centered care. Some studies have found that while family-based care improves self-management, it does not always translate into significant quality-of-life gains. For example, Saidi et al. reported that although family-centered care had a positive effect on self-management in patients with tuberculosis, it showed only limited improvement in their quality of life (32). This contrast may stem from differences in patient characteristics, such as the severity of the disease or the presence of comorbidities. Our study specifically targeted elderly patients with MDR-TB, a population that often faces unique challenges such as frailty, multiple comorbidities, and a higher susceptibility to psychological distress. These factors may have made the family collaboration care model more effective in addressing both physical and emotional needs, leading to more significant improvements in quality of life. Additionally, the duration and intensity of the intervention could account for variations in outcomes across studies. A previous study performed by Li et al. also reported that stratified management and personalized interventions could alleviate the burden of medication in MDR-TB populations (33). Our study utilized a personalized and sustained approach to family collaboration, which may have contributed to more significant improvements in self-management and disease perception compared to shorter or less intensive care.

From a practical perspective, the family collaboration care model could be incorporated into routine MDR-TB nursing management as an adjunctive behavioral and psychosocial support strategy. During hospitalization, nurses may identify a primary family caregiver, provide standardized education on MDR-TB, medication adherence, infection-control precautions, nutrition, and emotional support, and establish individualized care goals with both patients and caregivers. After discharge, telephone or outpatient follow-up could reinforce medication adherence, monitor self-management difficulties, and provide timely psychological support. Therefore, this model may be feasible within routine nursing workflows, particularly for elderly patients who require sustained family support during prolonged MDR-TB treatment.

This study has several limitations that should be considered when interpreting the results. First, the retrospective design and relatively small sample size limit our ability to establish causality between the family collaboration care model and improvements in disease perception, self-management, and quality of life. In future studies with a large sample size, we would consider multivariable regression adjustment or propensity score matching to control for potential confounders. Moreover, because group assignment was based on nursing records rather than randomization, there may be potential selection bias, and the findings of this study should be further validated in a future prospective study. Additionally, the lack of a long-term follow-up means that we cannot assess the sustainability of the benefits observed in the short term. Another limitation is the absence of detailed data on the severity of MDR-TB and comorbid conditions, which may have influenced the outcomes. Future prospective studies with larger sample sizes, long-term follow-up, and more comprehensive data collection are needed to further validate these findings and explore the mechanisms by which family involvement contributes to improved outcomes.

## Conclusion

In conclusion, this study indicates an association between the family collaboration care model and improved disease perception, self-management, and quality of life in elderly patients with MDR-TB, which supports its preliminary clinical application value. The involvement of family members is correlated with improvements in patients’ physical symptom management and psychological status, thereby potentially optimizing comprehensive treatment outcomes to a certain extent. The positive findings of this study suggest that integrating family-centered care into MDR-TB management may be conducive to favorable patient-related outcomes. Of note, as a retrospective observational study, this research cannot establish definitive causal relationships. Further high-quality studies are needed to verify the long-term effects of this care model and explore its generalizability in wider patient populations.

## Data Availability

The original contributions presented in the study are included in the article. Further inquiries can be directed to the corresponding author.
