# Supplementary material for: Effects of a family collaboration care model on disease perception, self-management, and quality of life in elderly patients with multidrug-resistant pulmonary tuberculosis: a retrospective study
Source: Front Med (Lausanne). 2026 Jun 3;13:1781223. doi: 10.3389/fmed.2026.1781223 (PMC13272382; doi:10.3389/fmed.2026.1781223)
Supplement: Supplementary file 1 [file Supplementary_file_1.docx]

Supplementary Table 1. The interaction effects (group × time) and effect size analyses for each outcome measure between the two groups.

|  | F statistic | Pvalue | Partial η² |
| --- | --- | --- | --- |
| Disease Perception Scores | 57.188 | <0.001 | 0.510 |
| Self-management Ability Scores | 54.618 | <0.001 | 0.498 |
| Quality of Life Scores | 81.314 | <0.001 | 0.597 |

Supplementary Table 2. Effect size analysis for between-group comparisons after the intervention.

|  | Effect size type | Effect size value | 95% CI |
| --- | --- | --- | --- |
| Disease Perception Scores | Cohen's d | −1.227 | [−1.796, −0.659] |
|  | Hedges' g | −1.211 | [−1.796, −0.659] |
| Self-management Ability Scores | Cohen's d | 1.882 | [1.254, 2.510] |
|  | Hedges' g | 1.856 | [1.254, 2.510] |
| Quality of Life Scores | Cohen's d | 2.531 | [1.828, 3.234] |
|  | Hedges' g | 2.496 | [1.828, 3.234] |
